# Supplementary material for: A diminutive perinate European Enantiornithes reveals an asynchronous ossification pattern in early birds
Source: Nat Commun. 2018 Mar 5;9:937. doi: 10.1038/s41467-018-03295-9 (PMC5838198; doi:10.1038/s41467-018-03295-9)
Supplement: Supplementary file 1 — Supplementary Information [file 41467_2018_3295_MOESM1_ESM.pdf]

Supplementary Note 1:

Elemental mapping

Faint ribbing in a geochemical precipitate visible in a yellowish stain close to the tail made us suspect the presence of plumage remains in MPCM-LH-26189. However, synchrotron tomography showed it to correspond to ribbon-like structures possibly more consistent in their morphology with vegetal material than with feathers. Nevertheless, the exceptional preservation of MPCM-LH-26189 means there was a possibility of soft tissue/biochemical information preservation that, whilst not visible to the naked eye, could be detectable as a chemical "ghost". Synchrotron Rapid Scanning X-ray Fluorescence (SRS-XRF) elemental imaging has previously shown the ability to detect dilute chemical remnants of soft tissues in many fossil specimens, even when no visible evidence of such tissues remain (e.g., refs 1-4). We, therefore, undertook SRS-XRF imaging of the specimen at the Stanford Synchrotron Radiation Lightsource (SSRL) wiggler beam line 6-2 at the Stanford Linear Accelerator Centre (SLAC, CA, USA). Detailed descriptions of SRS-XRF mapping applied to fossils are provided in recent previous publications[5-6] and so are only summarized here.

Experiments were operated with an incident beam energy of either 13.5 keV (flux calculated between 10^{10} and 10^{11} photons s-1) and a beam diameter set by a 50 micron pinhole (high Z) or 3.15 keV (flux ~10^{9} photons s-1) using a 25 micron pinhole (low Z). X-rays were detected using a single element Vortex silicon drift detector. For point quantification analyses, a full energy dispersive spectrum is collected for 100 live seconds. Energy dispersive spectra obtained from SSRL were fit using the PyMCA freeware[7] from fundamental parameters of the experiment using a Durango apatite (fluoroapatite) mineral standard with known element concentrations for calibration. SRS-XRF maps from SSRL were processed from the raw detector count raster files using a custom MATLAB computer script that converted the data array into viewable 8 bit tiff images clipped at various contrast percentiles.

The resulting elemental maps (Supplementary Fig. 2) showed that Al, Si, P, Fe, Cu, and Zn were associated with fossilised tissue, but were mostly constrained by the skeletal components thus indicating that little remains of the original soft-tissues. P is clearly elevated in the bones as would be expected (Supplementary Fig. 3) and resolves the bone morphology in extremely high fidelity, even in the area of the actual fossil

where it is hardly visible with the naked eye. Notably, P is present in concentrations that are almost identical to that seen in extant avian bone[8], suggesting that there has been minimal loss of this element into the surrounding matrix (Supplementary Table 2). However, Fe and Si do appear elevated in a diffuse pattern on the bottom half of the organism. A false colour composite image of Fe (red), Si (green), and P (blue) (Supplementary Fig. 4) shows that Fe and Si are highly correlated (yellow). P also appears to be weakly correlated with Fe and Si in this region. The distribution of these elements is somewhat similar to what the body outline would have been in life.

This diffuse distribution of Fe and P is consistent with the known mode of preservation of soft-tissue at the Las Hoyas site, which has been interpreted to be due to microbial/biofilm iron carbonate mineralisation and phosphatisation[9]. The distribution of silica is also consistent with mineralisation associated with microbial mats[10]. Unlike a wide range of other exceptionally preserved fossils studied via SRS-XRF, little organic sulphur is associated with this fossil integument (Supplementary Fig. 2). Sulphur X-ray absorption spectroscopy (Supplementary Fig. 5) shows no difference in sulphur speciation between matrix and fossil, consisting predominantly of sulphate (~2481.75 eV), although a minute amount of reduced sulphur may be resolved (~2473.25 eV). This indicates that the soft tissue in this case is almost entirely replaced, such that some details of integument distribution are still visible, but remnants of original biochemistry are at limits of detection for the techniques applied here.

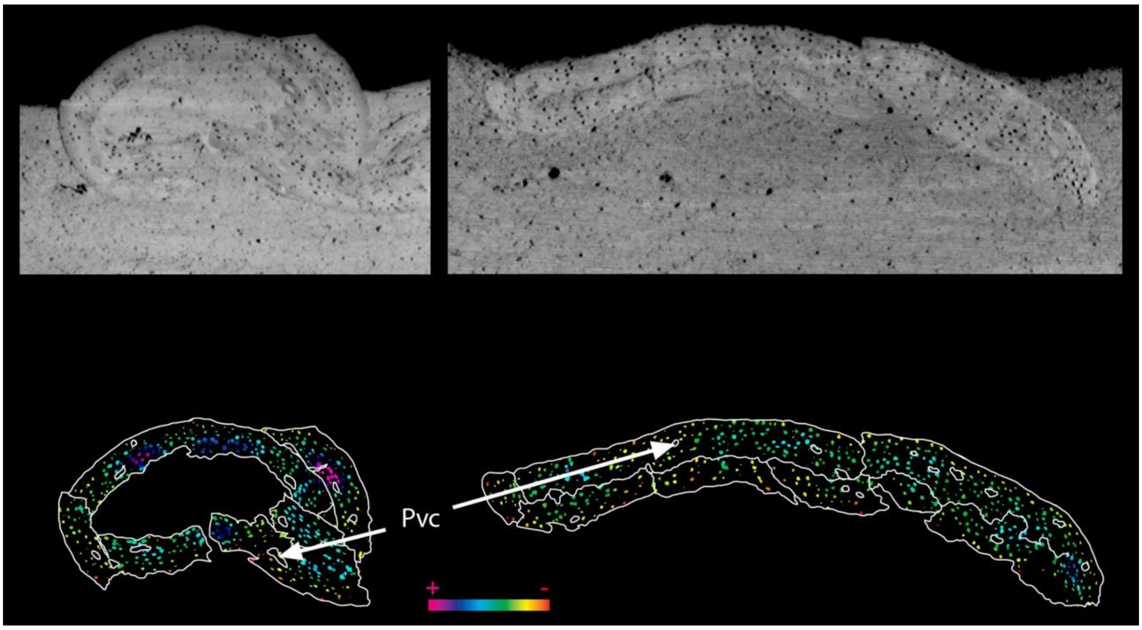

Supplementary Figure 1 | Map of the bone cell lacunae in the tibia and radius of MPCM-LH-26189 b. Colour coding shows the gradation between the densest (+) and least dense (-) regions. Abbreviation: Pvc, primary vascular canal.

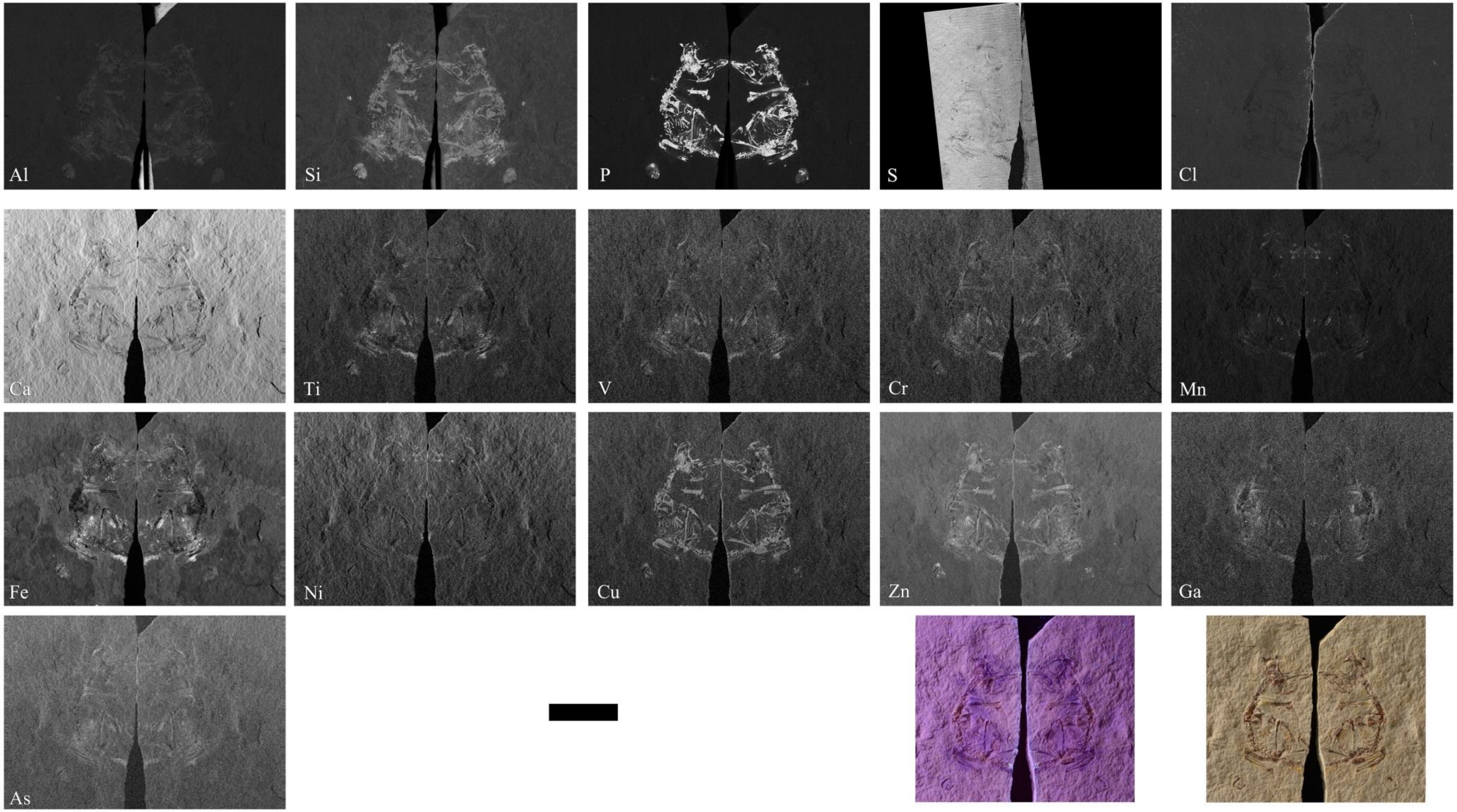

Supplementary Figure 2 | SRS-XRF elemental maps of MPCM-LH-26189. Low Z maps (upper row), high Z maps (lower rows), optical photograph under ultraviolet light (lowest row, right), optical photograph under normal light (lowest row, far right). Scale bar = 20 mm.

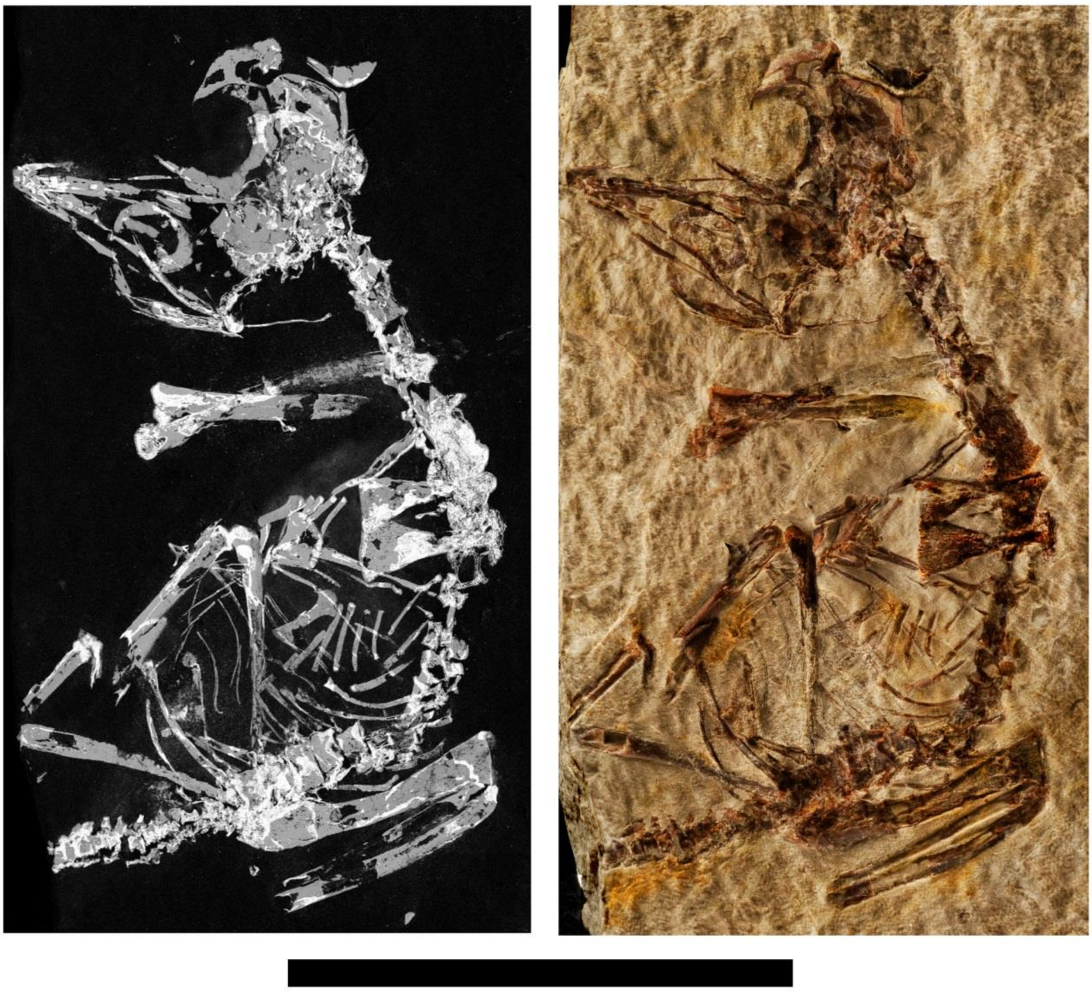

Supplementary Figure 3 | Overlay of the phosphorus SRS-XRF maps (left) and optical photographs (right) of the slab and counterslab of MPCM-LH-26189. This provides a visual representation of the combined preserved skeletal elements (partial transparency applied to MPCM-LH-26189 b). The sternal elements are particularly well resolved in the elemental map. Scale bar = 20 mm.

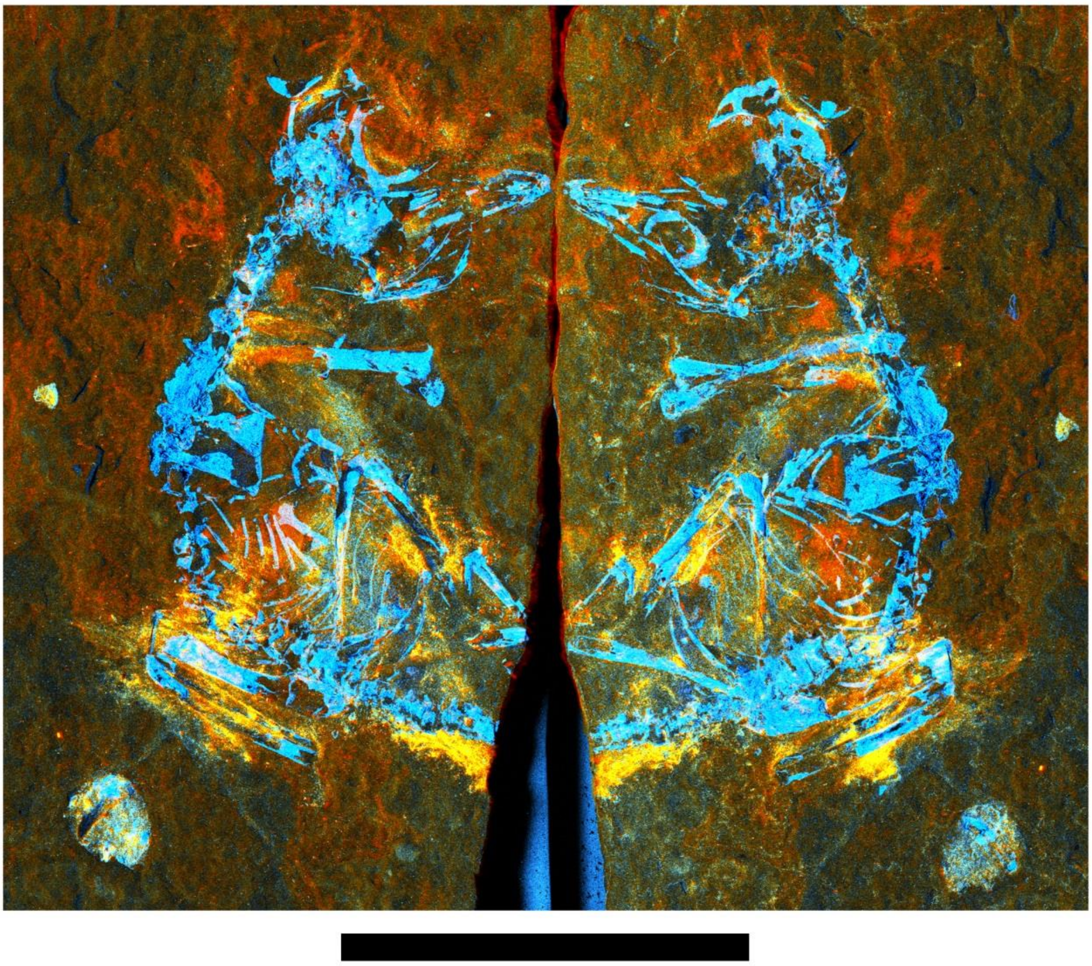

Supplementary Figure 4 | False colour composite image of SRS-XRF elemental maps of MPCM-LH-26189. Red=Fe, Green=Si, Blue=P. This image is not representative of true relative concentrations, but is designed to best illustrate the distributions of these elements. Fe and Si correlate in some areas (yellow regions) potentially representing remnant soft tissue. Scale bar = 20 mm.

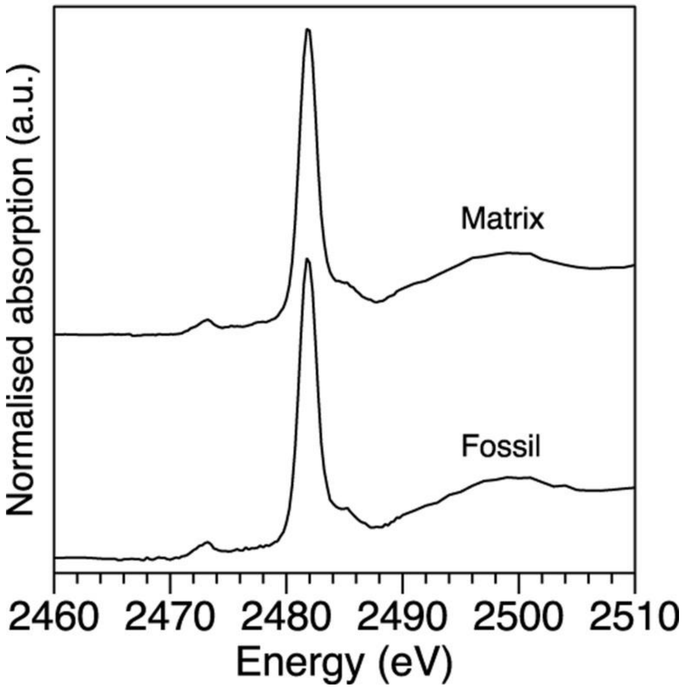

Supplementary Figure 5 | Sulphur X-ray Absorption Near Edge Structure (XANES) of MPCM-LH-26189. These spectra show no difference in sulphur speciation between the matrix and a location within the abdomen of the fossil (as is suggested by the SRS-XRF map of S). The dominant peak at 2481.75 eV shows that the sulphur in this specimen is mostly sulphate. A small peak at ~2473.25 eV shows that there is some reduced (organic) sulphur, but it is not associated with any potential remnant of soft tissue.

	Iberomesornis (MPCM-LH-22)	*Concornis* (MPCM-LH-2814)	MPCM-LH-26189
**Humerus/ulna**	0.99	0.98	1.07
**Humerus/radius**	0.98	1.08	1.22
**Humerus/femur**	1.04	1.29	0.98

Supplementary Table 1 | Ratio of the length of the humerus against that of other long bones in the birds from Las Hoyas. The brachial index (humerus length/ulna length) of MPCM-LH-26189is large compared to that of most other Enantiornithes, but allometric changes occurring during growth are suspected in juvenile Enantiornithes, and have been demonstrated in some fossil birds. For instance, it has been demonstrated that the humerus of the Early Cretaceous *Sapeornis* is longer than the ulna in the smallest and youngest specimens but shorter in larger and older individuals[11]. This is similar to what can be seen in a number of modern birds[12-14].

	Al	Si	P	S	Cl	Ca	Ti	Mn	Fe	Ni	Cu	Zn	Ga	As	Se	Ce
**Matrix**	9903	8141	598	4915	247	38.95%	509	74	2275	23	5	39	2	5	/	/
**Skull/Head**	2.7%	1.94%	15.10%	8121	283.9	62.69%	475	63	1167	39	63	114	1	7	11	490
**Gut/Stomach**	1.85%	2.24%	1.14%	5075	273	44.19%	1263	28	1.6%	38	55	184	8	12	8	725
**Humerus**	-	-	-	-	-	34.82%	188	39	710	15	34	87	1	6	10	237
**Tail**	-	-	-	-	-	43.90%	785	115	2271	29	4	40	2	5	1	3
**Pelvis**	2.88%	1.97%	16.06%	9420	259	-	-	-	-	-	-	-	-	-	-	-

Supplementary Table 2 | Synchrotron XRF Energy Dispersive Spectroscopic quantification of MPCM-LH-26189. Values are in parts per million (ppm) except where indicated in weight %. Errors estimated at ~10% absolute value. / = element not detected; - = Data not obtained.

Supplementary References

1. Bergmann, U. *et al*. *Archaeopteryx* feathers and bone chemistry fully revealed via synchrotron imaging. *Proc. Natl. Acad. Sci. USA* **107**, 9060–9065 (2010).
2. Edwards, N. P. *et al*. Infrared mapping resolves soft tissue preservation in 50 million year-old reptile skin. *Proc. Roy. Soc. B* **278**, 3209–3218 (2011).
3. Edwards, N. P. *et al*. Mapping prehistoric ghosts in the synchrotron. *Appl. Phys. A* **111**, 147–155 (2013).
4. Manning, P. L. *et al*. Synchrotron-based chemical imaging reveals plumage patterns in a 150 million year old early bird. *J. Anal. At. Spectrom.* **28**, 1024–1030 (2013).
5. Anné, J. *et al*. Synchrotron imaging reveals bone healing and remodelling strategies in extinct and extant vertebrates. *J. Roy. Soc. Interface* **11**, 20140277 (2014).
6. Edwards, N. P. *et al*. Leaf metallome preserved over 50 million years. *Metallomics* **6**, 774–782 (2014).
7. Solé, V. A., Papillon, E., Cotte, M., Walter, P. & Susini, J. A multiplatform code for the analysis of energy-dispersive X-ray fluorescence spectra. *Spectrochim. Acta B* **62**, 63–68 (2007).
8. Liu, X. *et al*. P and trace metal contents in biomaterials, soils, sediments and plants in colony of red-footed booby (*Sula sula*) in the Dongdao Island of South China Sea. *Chemosphere* **65**, 707–715 (2006).
9. Guerrero, M. del C., López-Archilla, A. I., & Iniesto, M. In *Las Hoyas: a Cretaceous Wetland* (eds Poyato-Ariza, F. J. & Buscalioni, Á. D.) 220–228 (F. Pfeil, 2016).
10. Konhauser K. O. & Ferris, F. G. Diversity of iron and silica precipitation by microbial mats in hydrothermal waters, Iceland: implications for Precambrian iron formations. *Geology* **24**, 323–326 (1996).

11. Pu, H. *et al*. A new juvenile specimen of *Sapeornis* (Pygostylia: Aves) from the Lower Cretaceous of Northeast China and allometric scaling of this basal bird. *Paleontol. Res.* **17**, 27–38 (2013).
12. Graul, W. Zur Entwicklung von *Vanellus cristatus*. *Arch. Naturgesch.* **73**, 153–180 (1907).
13. Latimer, H. B. Postnatal growth of the chicken skeleton. *Am. J. Anat.* **40**, 1–57 (1927).
14. Maillard, J. Recherches embryologiques sur *Catharacta skua* Brünn. (ptérylose et ossification). *Rev. Suisse Zool.* **55**, 1–114 (1948).
